# Schizophrenia-associated differential DNA methylation in brain is distributed across the genome and annotated to *MAD1L1*, a locus at which DNA methylation and transcription phenotypes share genetic variation with schizophrenia risk

**DOI:** 10.1038/s41398-022-02071-0

**Published:** 2022-08-20

**Authors:** Brandon C. McKinney, Lora L. McClain, Christopher M. Hensler, Yue Wei, Lambertus Klei, David A. Lewis, Bernie Devlin, Jiebiao Wang, Ying Ding, Robert A. Sweet

**Affiliations:** 1 Department of Psychiatry, University of Pittsburgh, Pittsburgh, PA USA; 2 Translational Neuroscience Program, Department of Psychiatry, University of Pittsburgh, Pittsburgh, PA USA; 3 Department of Biostatistics, University of Pittsburgh, Pittsburgh, PA USA; 4 Department of Neurology, University of Pittsburgh, Pittsburgh, PA USA

**Keywords:** Schizophrenia, Pathogenesis

## Abstract

DNA methylation (DNAm), the addition of a methyl group to a cytosine in DNA, plays an important role in the regulation of gene expression. Single-nucleotide polymorphisms (SNPs) associated with schizophrenia (SZ) by genome-wide association studies (GWAS) often influence local DNAm levels. Thus, DNAm alterations, acting through effects on gene expression, represent one potential mechanism by which SZ-associated SNPs confer risk. In this study, we investigated genome-wide DNAm in postmortem superior temporal gyrus from 44 subjects with SZ and 44 non-psychiatric comparison subjects using Illumina Infinium MethylationEPIC BeadChip microarrays, and extracted cell-type-specific methylation signals by applying tensor composition analysis. We identified SZ-associated differential methylation at 242 sites, and 44 regions containing two or more sites (FDR cutoff of *q* = 0.1) and determined a subset of these were cell-type specific. We found mitotic arrest deficient 1-like 1 (*MAD1L1*), a gene within an established GWAS risk locus, harbored robust SZ-associated differential methylation. We investigated the potential role of *MAD1L1* DNAm in conferring SZ risk by assessing for colocalization among quantitative trait loci for methylation and gene transcripts (mQTLs and tQTLs) in brain tissue and GWAS signal at the locus using multiple-trait-colocalization analysis. We found that mQTLs and tQTLs colocalized with the GWAS signal (posterior probability >0.8). Our findings suggest that alterations in *MAD1L1* methylation and transcription may mediate risk for SZ at the *MAD1L1*-containing locus. Future studies to identify how SZ-associated differential methylation affects *MAD1L1* biological function are indicated.

## Introduction

Schizophrenia (SZ) is a severe neuropsychiatric disorder with complex etiology. Heritability estimates for SZ from twin studies are consistently ~80% [1], thus suggesting a substantial genetic contribution to its etiology. Genome-wide association studies (GWAS) have identified many single-nucleotide polymorphisms (SNPs) associated with SZ, although each SNP has only a small effect on risk for the disorder [2]. A recent large-scale GWAS meta-analysis identified SNPs at 270 distinct genetic risk loci [3]. Heritability estimates from GWAS fall short of those predicted by twin studies, thus suggesting that other forms of genetic variation contribute to risk for SZ. Indeed, recent studies have found a high burden of both rare SNPs and rare copy number variants in individuals diagnosed with SZ [4, 5].

SZ-associated SNPs often alter local DNA methylation (DNAm) [6–8]. DNAm, the addition of a methyl group to a cytosine in DNA, stably affects gene expression via interaction with transcription factor binding [9]. DNAm is associated with both increased and decreased gene expression as well as other forms of gene regulation, including splicing and alternative promoter usage [9–11]. Changes in DNAm, acting through effects on gene expression, represent one potential mechanism by which SZ-associated SNPs can confer risk.

The superior temporal gyrus (STG) is a region of the brain critical for auditory processing. In individuals with SZ, altered STG function is associated with auditory verbal hallucinations and impaired auditory sensory processing. Impaired auditory processing further contributes to phonologic dyslexia and difficulty recognizing and expressing spoken emotional tone (prosody) in SZ [12].

In this study, we investigated genome-wide SZ-associated differential methylation in the STG. To this end, we used Illumina Infinium MethylationEPIC BeadChip microarrays (EPIC arrays) to measure DNAm at ~850,000 sites across the genome in the STG from 44 subjects with SZ and 44 non-psychiatric comparison (NPC) subjects. We applied tensor composition analysis (TCA) to extract cell-type-specific DNAm signals from brain tissue-level data. These analyses identified several genes that harbored cell-type-specific differences in DNAm between SZ and NPC subjects including mitotic arrest deficient 1-like 1 (*MAD1L1*), a gene within one of the 270 SZ risk loci identified in the largest GWAS study to date and one of 130 genes thought highly likely to explain the association between GWAS loci and SZ [3]. Our subsequent analyses focused on *MAD1L1*. To gain insight into the potential role of *MAD1L1* DNAm in conferring SZ risk, we identified methylation and transcript quantitative trait loci (mQTLs and tQTLs) for *MAD1L1* in postmortem cerebral cortex using publicly available data [7, 13] and performed multiple-trait-colocalization (MOLOC) analysis to assess for statistical colocalization [14], or shared genetic traits, using the methylation and transcription phenotypes and the GWAS signal at the *MAD1L1*-containing locus.

## Materials and methods

### Postmortem brains

Tissue was obtained from postmortem brains recovered and processed as described previously [15, 16]. Briefly, brains were retrieved during routine autopsies at the Allegheny County Medical Examiner’s Office, Pittsburgh, PA, USA, following informed consent from next-of-kin. An independent committee of experienced clinicians made consensus Diagnostic and Statistical Manual of Mental Disorders, Fourth Edition diagnoses, or determined the absence thereof, based on clinical records and collateral history obtained via structured interviews with surviving relatives [17]. The right hemisphere was blocked coronally and the resultant slabs snap frozen and stored at −80 °C. Slabs containing the STG were identified and the STG was removed as a single block from each of the slabs in which it was present. Samples containing all six cortical layers of STG (planum temporale), but excluding the adjacent white matter, were harvested. All procedures were approved by the University of Pittsburgh Committee for the Oversight of Research and Clinical Training Involving Decedents and the Institutional Review Board for Biomedical Research.

### Cohort membership

The cohort comprised 44 subjects with either SZ (*N* = 31) or schizoaffective disorder (*N* = 13), and 44 NPC subjects. Subjects diagnosed with SZ and schizoaffective disorder were grouped together for analysis, and referred to as SZ subjects, or the SZ group. In this study, as in our previous studies, we found that the diagnoses do not differ with respect to DNAm [18]. Each subject in the SZ group was matched with one NPC subject for sex, hemisphere, and as closely as possible for postmortem interval (PMI), age, and other characteristics (Table 1 and Supplementary Table 1).

**Table 1:** Cohort characteristics.

Group	NPC	SZ
Number	44	44
Sex	32 M, 12 F	31 M, 13 F
Race	35 W, 8 B, 1 O	32 W, 12 B
Age (years)	48.25 ± 13.82	47.48 ± 13.88
PMI (h)	17.41 ± 5.89	18.32 ± 7.05
pH	6.70 ± 0.28	6.56 ± 0.31

Data for continuous variables are presented as group average ± standard deviation.

*B* black, *F* female, *M* male, *NPC* non-psychiatric comparison, *O* other (Asian Indian), *PMI* postmortem interval, *SZ* schizophrenia, *W* white.

### DNA preparation and bisulfite conversion

DNA (~10 μg) was isolated from STG gray matter (~20 mg) using AllPrep DNA/RNA/Protein Mini Kit (Qiagen, Valencia, CA, USA) and bisulfite was converted using EZ-96 DNA Methylation Kit (Zymo Research, Irvine, CA, USA), both as per the manufacturer’s protocol.

### DNA methylation arrays

DNAm is the addition of a methyl group to a cytosine in DNA. DNAm is observed within the context of cytosine-phosphate-guanine dinucleotides (CpGs), most commonly, but also within the context of cytosine-phosphate-H dinucleotides (CpHs, where H = cytosine, adenine, or thymine) [19, 20]. CpGs and CpHs are referred to as “DNAm sites” or “sites” in this manuscript. DNAm was measured at 866,091 sites using MethylationEPIC BeadChip Infinium array (EPIC array; Illumina, San Diego, CA, USA) as per the manufacturer’s protocol [21, 22]. A *β*-value, the proportion of a particular site that is methylated in a DNA sample, was determined for each site by taking the ratio of the methylated to unmethylated signal, using the formula: *β* value = intensity of the methylated signal/(intensity of the unmethylated signal + intensity of the methylated signal + 100). A 96-entry EPIC array was filled with samples from the 88 subjects, including replicate samples from eight subjects. Data are available for download from Gene Expression Omnibus (GEO; GSE144910).

### Data processing and filtering

Data analyses were performed using the R software environment (www.r-project.org).

Color adjustment and background correction were performed using the bgAdjust2C method [23]. Normalization was performed using the *preprocessQuantile* function in the R package *minfi* [24]. The initial dataset comprised data from 1,051,815 probes corresponding to 866,091 DNAm sites for each subject. Multidimensional scaling (MDS) was used to visualize the degree of similarity among samples [25]. Prior to data filtering, samples were segregated strongly by sex in MDS space (Supplementary Fig. 1A). Data filtering involved removing all data points associated with a probe if the probe failed detection as indicated by a median detection *p* value >0.01 (probes corresponding to 12,350 DNAm sites), cross-reacted with multiple genomic regions (probes corresponding to 39,269 DNAm sites), contained an SNP within its binding site (probes corresponding to 27,395 DNAm sites), or interrogated a DNAm site on a sex chromosome (probes corresponding to 18,628 DNAm sites). Data from probes corresponding to 768,449 DNAm sites remained for downstream analysis (Supplementary Fig. 2). After data filtering, MDS using data from the 3000 most variable sites was performed and samples were no longer segregated by sex (Supplementary Fig. 1B), but segregation by race (Supplementary Fig. 1C) and age (Supplementary Fig. 1D, E) became evident. The replicate sample pairs from each of the eight subjects from which replicate samples were collected and assayed co-segregated in MDS space (Supplementary Fig. 1F), thus demonstrating the reproducibility of our approach. The *β*-values for each replicate pair were averaged for the downstream analyses.

### Differential DNA methylation

Linear regression was used to identify differentially methylated sites (DMSs). DNAm, in the form of preprocessed and normalized *β*-values, was the dependent variable and diagnosis was the independent variable. Race, age, and PMI were included as covariates in the analysis. The MDS analysis described above supported the inclusion of race as a covariate. Most subjects in this cohort self-identified as either white or black; however, one subject self-identified as Asian Indian and, consistent with known genetic architecture [26], clustered with the subjects of European ancestry (Supplementary Fig. 1C) and was thus combined with the subjects that self-identified as white for analyses. The inclusion of age as a covariate is supported by the MDS analysis as well as existing literature that shows age has a robust effect on DNAm [27–29]. Though samples did not segregate by PMI in MDS space (data not shown), it was included as a covariate because the stability of many molecular measures has been found to be particularly sensitive to PMI [30, 31], and to maintain consistency with our previous study in which it was included as a covariate in our primary analyses [18].

Differential methylated regions (DMRs) were identified using the R package DMRcate [32]. DMRcate uses an approach based on tunable kernel smoothing of the differential methylation signal across the genome obtained in the site-based differential DNAm analysis described above. A Benjamini–Hochberg corrected false discovery rate (FDR) <0.1 for the smoothed signal was considered significant. Then regions with a maximum of 1000 base pairs containing at least two such significant sites were defined as DMRs.

### Neuron and glia proportion estimates

The proportion of neurons and glia in each sample was estimated with CETS, an R package that uses *β* values from cell-type-specific sites to generate the estimation [33].

### Neuron- and glia-specific differential DNA methylation

The CETS-estimated proportions of neurons and glia for each subject and TCA [34] were used to estimate the subject-level neuron- and glia-specific *β* values for each DNAm site and detect sites at which DNAm differs between subjects with SZ and NPC subjects. The cell-type proportions were refit in TCA version 1.1.0 and the cell-type-specific differential methylation analysis is done with default TCA settings and adjusted for age, race, and PMI assuming they affect tissue-level DNAm.

### Relating DNA methylation and gene transcription to GWAS signal at the MAD1L1-containing SZ risk locus

#### Fine mapping

GWAS [3] have established signals of association between SNPs in a locus containing *MAD1L1*. FINEMAP, a software package that evaluates various potential causal variant configurations to produce posterior probabilities of association (PPA) that a given SNP or set of SNPs can account for the GWAS signal [35], was used to localize the GWAS signal to a set of plausible causal SNPs at this locus.

#### Colocalization analysis

SNPs that associate with methylation levels of cytosine in DNA, or mQTLs, at the *MAD1L1* locus were identified using data from Jaffe et al. [7]. Likewise, PsychENCODE data [13] were used to identify SNPs that associate with the abundance of a gene transcript, or tQTLs. To assess for colocalization among GWAS signal, mQTLs, and tQTLs at the *MAD1L1* locus, PPA was computed for each SNP with GWAS *p* value <5 × 10^−15^ (FINEMAP 0.002 < PPA < 0.253) using multi-trait colocalization (MOLOC, 78). A PPA >0.8 was considered evidence of colocalization (see Supplementary methods for details).

## Results

### SZ-associated differential DNA methylation was identified at many individual sites and genomic regions, including within *MAD1L1*

**Fig. 1:**
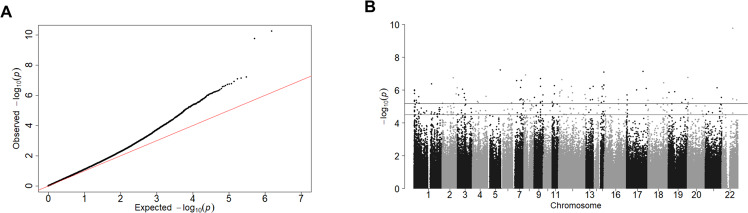
SZ-associated differential methylation. **A** Probability plot showing that the analysis for sites at which DNAm differed between SZ and NPC subjects is enriched in small *p* values compared to what would be expected by chance. The *y* = *x* line represents the distribution of *p* values that would be expected by chance. **B** Manhattan plot showing that the DNAm differed between subjects with SZ and NPC subjects at many DNAm sites, and the sites were distributed across many autosomes. The horizontal lines represent FDR cutoff of *q* = 0.1 (bottom) and *q* = 0.05 (top). DNAm DNA methylation, SZ schizophrenia, NPC non-psychiatric comparison, FDR false discovery rate.

DNAm differed between subjects with SZ and NPC subjects at more sites than would be expected by chance (Fig. 1A). DNAm differed at 242 sites between subjects with SZ and NPC subjects with an FDR cutoff of *q* = 0.1 (Table 2). Of these 242 DMSs, DNAm differed at 101 sites with an FDR cutoff of *q* = 0.05. No global differences in DNAm were identified between SZ and NPC subjects (Supplementary Fig. 3). The sites at which DNAm differed between subjects with SZ and NPC subjects were broadly distributed across all autosomes (Fig. 1B).

**Table 2:** Differentially methylated sites in SZ.

Site	DNAm difference (SZ-NPC; *β* values)	*q* value	Gene
cg01712700	−0.032	4.55E–05	CAPN10
cg13532802	−0.041	6.82E–05	
cg04020590	−0.032	0.013	GRTP1
cg08847417	−0.023	0.013	ZNF827
cg05621596	−0.018	0.013	GRAMD4
cg25079492	−0.028	0.016	CLEC16A
cg24941703	−0.028	0.017	MAD1L1
cg07748741	−0.012	0.017	UBTD1
cg23379913	−0.026	0.017	AKAP1
cg04011474	−0.028	0.018	
cg22945957	−0.028	0.018	PSTPIP1
cg08692211	−0.021	0.018	MEIS2
cg22519912	−0.027	0.022	PSD2
cg06050636	−0.035	0.024	S100A13
cg22689280	−0.029	0.024	
cg02478836	−0.022	0.024	TBC1D22A
cg26981306	−0.029	0.024	KIAA0892
cg13913915	−0.032	0.024	MSI2; MSI2
cg23453794	−0.032	0.024	MERTK
cg22348992	−0.016	0.024	CHRNA4
cg19261949	−0.024	0.024	ZMAT5
cg12349571	−0.018	0.024	TLE3
cg24158028	−0.028	0.024	STK32C
cg21265339	−0.023	0.024	
cg02289653	−0.025	0.024	EPB41L1
cg18282392	−0.019	0.024	GALNT7
cg25459000	−0.018	0.024	SYNGR1
cg19418922	−0.027	0.025	EXT2
cg02722031	−0.028	0.026	HERC3
cg01606571	−0.027	0.026	HADHA
cg03449456	−0.020	0.026	PRDM16
cg17601209	−0.032	0.026	PRDM16
cg12937501	−0.025	0.029	
cg10699522	−0.028	0.029	DST; LOC101930010
cg24920126	−0.022	0.031	PPP1R3G
cg23200394	−0.033	0.031	GLI2
cg12446793	−0.028	0.033	
cg25894668	−0.021	0.033	SLC3A2
cg07348768	−0.015	0.033	PRDM16
cg16901627	−0.020	0.033	COPE
cg00387200	−0.027	0.033	
cg21620968	−0.028	0.035	COPS7B
cg25211200	−0.026	0.035	MRVI1
cg20402747	−0.020	0.035	TBC1D16
cg13523224	−0.032	0.035	CFAP99
cg06022867	−0.043	0.038	
cg15044372	−0.025	0.039	
cg24318537	−0.024	0.039	UNC119B
cg21408848	−0.026	0.040	IQSEC1
cg12713481	−0.027	0.040	
cg21946195	−0.034	0.040	ATOH8
cg09815962	−0.027	0.041	EIF2C2
cg14608424	−0.025	0.041	ABR
cg24512544	−0.021	0.041	EIF2C2
cg17134838	−0.023	0.041	
cg18329758	−0.027	0.041	WWC1
cg13689085	−0.026	0.041	TCF7
cg16266918	−0.024	0.041	PDXK
cg23634532	−0.023	0.042	OGDH
cg16433632	−0.014	0.043	RAMP1
cg14517390	−0.019	0.043	ACSBG1
cg07605200	−0.026	0.043	
cg19788036	−0.026	0.043	
cg02347483	−0.022	0.043	CCDC101
cg01433955	−0.028	0.043	
cg00686823	−0.024	0.043	TPRA1
cg08419879	−0.018	0.043	PLEKHG1
cg03595140	−0.027	0.043	FNBP1
cg21785920	−0.038	0.043	LBP
cg25298833	−0.027	0.043	RGMA
cg01287037	−0.028	0.043	
cg03932760	−0.022	0.043	ARRB1; MIR326
cg14372037	−0.030	0.043	SORCS2
cg15165927	−0.024	0.043	NKD2
cg25601830	−0.022	0.043	AKR7A2
cg01203812	−0.026	0.043	PRDM16
cg17803589	−0.023	0.043	SLC19A1
cg15845746	−0.012	0.043	TMEM177
cg27151770	−0.022	0.045	ZNF423
cg26520908	−0.028	0.045	PRDM16
cg04500745	−0.017	0.045	MAPK8IP3
cg14020176	−0.020	0.045	SLC9A3R1
cg04633409	0.019	0.045	TWF1
cg07597386	−0.007	0.045	PRDM16
cg00159552	−0.018	0.045	TBC1D22A
cg26632239	−0.014	0.045	CTDP1
cg13136596	−0.034	0.048	MSI2
cg24883899	−0.018	0.048	APC2
cg16023894	−0.030	0.048	EPHB2
cg16011164	−0.028	0.048	MIR4656; AP5Z1
cg00162902	−0.019	0.048	FAM184A
cg09925572	−0.025	0.048	TFCP2
cg17529670	−0.022	0.048	BCR
cg01123449	−0.026	0.048	HHIPL1
cg18783374	−0.024	0.048	MSI2
cg16622899	0.020	0.048	MAFK
cg08256119	−0.025	0.048	MSI2
cg14297573	−0.032	0.049	PFKP
cg12590902	−0.022	0.049	ERI3
cg06317803	0.018	0.049	
cg05068943	−0.019	0.049	
cg24338094	−0.022	0.052	PLXNA1
cg22649529	−0.035	0.053	TECR
cg19736604	−0.024	0.053	TNXB
cg01952185	0.019	0.053	
cg05501958	−0.011	0.053	APOE
cg26409376	−0.032	0.053	
cg04618897	−0.028	0.053	KIAA0415
cg08209664	−0.020	0.053	ST3GAL1
cg24484600	−0.022	0.053	GDPD5
cg07987705	−0.021	0.053	RGMA
cg15728120	−0.018	0.053	CENPT
cg09788030	−0.025	0.053	
cg08425757	−0.012	0.055	TRAPPC9
cg08196145	−0.018	0.055	
cg07463740	−0.025	0.055	
cg11301187	−0.025	0.055	KIAA0195
cg27214458	−0.012	0.055	MRGPRF; MRGPRF-AS1
cg23879743	−0.019	0.055	
cg03629926	−0.020	0.055	ANGPTL4
cg21126828	−0.027	0.056	RAI1
cg20108328	−0.024	0.056	C21orf70
cg23564627	−0.023	0.057	PEMT
cg07504768	−0.022	0.057	MTSS1L
cg22548266	−0.026	0.058	SPOCK2
cg04792024	−0.024	0.059	TMEM120A
cg13259703	−0.022	0.059	
cg19177744	−0.024	0.059	
cg12985235	−0.010	0.059	MPND
cg15343406	−0.038	0.061	
cg04594439	−0.025	0.061	PASK
cg17282060	−0.012	0.061	ARHGAP22
cg17196564	−0.020	0.062	
cg13175786	−0.022	0.062	PRDM16
cg09214323	−0.024	0.062	RNU6-2; KIF1B
cg08213909	−0.026	0.063	MCC
cg17736422	−0.034	0.063	PRDM16
cg26201596	0.026	0.065	
cg15748271	−0.020	0.065	TRIM8
cg17320669	−0.026	0.066	CAPN2
cg26301507	−0.018	0.068	SLC25A20
cg05141465	−0.026	0.069	CHST10
cg03950655	−0.021	0.069	ROR1
cg03628962	−0.021	0.069	RGMA
cg13821176	−0.029	0.069	TRIB1
cg15365305	−0.021	0.070	SMARCA2
cg10225499	−0.031	0.070	EZR
cg15412087	−0.024	0.070	OAF
cg03957687	−0.024	0.070	CENPT
cg01053681	−0.027	0.070	ZMIZ1
cg00726470	−0.017	0.071	
cg06520014	−0.016	0.071	
cg18069081	−0.025	0.072	GPR39
cg03272941	−0.020	0.073	RHOJ
cg26122413	−0.019	0.075	INF2
cg05071292	−0.013	0.075	LOC728613
cg11197533	−0.025	0.076	IFT122
cg26000554	−0.023	0.076	MOSC2
cg21036560	−0.024	0.076	PGBD5
cg12441066	−0.026	0.076	MSI2
cg08589214	−0.023	0.076	CAPN10
cg05878289	−0.023	0.076	SORCS2
cg04964562	−0.016	0.076	PLCG1
cg25619978	0.017	0.076	TRPC7
cg03747028	0.011	0.077	TAF12
cg06847567	0.019	0.078	
cg02133510	−0.023	0.078	TNXB
cg12863924	−0.026	0.078	
cg24379495	−0.021	0.078	SLC1A2
cg17823326	−0.017	0.079	NUBPL
cg26489368	−0.023	0.079	NKD2
cg20988960	−0.030	0.079	PRDM16
cg25456772	0.017	0.079	RAB3IP
cg04844692	−0.021	0.079	C12orf49
cg08067895	−0.024	0.079	CDX1
cg13153666	−0.022	0.080	
cg14597213	−0.021	0.082	AHCYL1
cg09792192	−0.017	0.082	AHCYL2
cg25122824	−0.017	0.082	MAD1L1
cg07410783	−0.025	0.082	CLEC16A
cg15763706	−0.026	0.082	SRGAP3
cg13547132	−0.020	0.082	
cg02986801	−0.019	0.083	ST3GAL1
cg11569621	−0.019	0.084	
cg26051775	−0.019	0.084	CAPN2
cg24986651	−0.023	0.084	LPIN1
cg01419991	−0.022	0.085	TRIB1
cg02743070	−0.019	0.085	ZMIZ1
cg05808227	−0.024	0.085	
cg06714043	−0.040	0.085	
cg09509365	−0.020	0.085	PRDM16
cg05321174	−0.014	0.086	PTK2B
cg00305491	−0.026	0.088	WWC1
cg22738000	−0.014	0.088	RASSF4
cg15900987	−0.016	0.088	BGLAP
cg07580832	−0.021	0.088	MSI2
cg19710386	−0.022	0.088	PTPRF
cg24699097	−0.016	0.089	RAB11FIP4
cg09761288	−0.023	0.089	
cg09255521	−0.033	0.090	
cg17877405	−0.025	0.090	CST3
cg15232718	−0.028	0.090	UBTD1
cg22177068	−0.028	0.090	ATP13A4-AS1; ATP13A4
cg17214089	−0.024	0.090	GLUL
cg00659252	−0.017	0.090	ASPH
cg13904892	−0.019	0.090	C15orf62; DNAJC17
cg08333580	−0.019	0.090	SLC1A2
cg26654807	0.015	0.091	ZMIZ1
cg11047279	−0.021	0.092	
cg18773993	−0.022	0.093	ABCA4
cg07380086	−0.024	0.093	CHN1
cg05912181	−0.019	0.093	LOC100506497
cg05747038	−0.018	0.093	GLIS3
cg17505776	−0.015	0.093	ITSN1
cg09676376	−0.023	0.094	ZNF385A
cg21184699	−0.020	0.094	FAM120A
cg24186251	−0.023	0.094	SH3RF3
cg13721930	−0.018	0.094	
cg15395783	−0.022	0.096	HEYL
cg21148160	−0.028	0.096	PAPLN
cg10782534	0.015	0.097	
cg23919411	−0.025	0.097	SEC14L4
cg12480689	−0.024	0.097	PFKFB2
cg16028934	−0.008	0.097	TP53BP2
cg21049762	−0.027	0.097	TCIRG1
cg10589385	0.056	0.097	SETDB1
cg06873567	−0.023	0.098	
cg13461192	−0.024	0.098	RHOQ
cg12128274	0.028	0.098	CNOT4
cg13691436	−0.023	0.098	FRMD4A
cg02276845	−0.006	0.098	STIM1
cg10051022	−0.015	0.098	FGGY
cg17876641	−0.025	0.098	KIF21B
cg19705197	−0.021	0.098	PFKFB3
cg03718662	−0.020	0.098	RASAL2
cg25307778	−0.021	0.098	ERI1
cg13302567	−0.024	0.098	MAD1L1
cg25674846	−0.018	0.098	LOC100506603; ANGEL1
cg00104333	−0.019	0.098	LGI1
cg07303829	−0.019	0.098	PPP6R2
cg02752163	0.043	0.098	
cg17931415	−0.028	0.098	MSI2

The 242 sites at which DNAm differed between SZ and NPC subjects with the FDR cutoff of *q* = 0.1 (adjusted for age, race, and PMI) are listed in the table.

*DNAm* DNA methylation, *NPC* non-psychiatric comparison, *PMI* postmortem interval, *SZ* schizophrenia.

DNAm is known to differ markedly between neurons and glia (36), and detection of DNAm differences between groups in tissue with multiple cell types can be confounded by cell composition. In STG samples studied here, neuronal proportion did not differ between subjects with SZ and NPC subjects (SZ = 0.46 ± 0.05; NPC = 0.46 ± 0.04; *p* = 0.50) (Supplementary Table 2A), and we have previously shown that pyramidal neuron number in layer 3 of this brain region did not differ between subjects with SZ and NPC subjects (37). After adjusting for neuron proportion, DNAm differed at 256 sites between SZ and NPC subjects with the FDR cutoff of *q* = 0.1 (Supplementary Table 2B). Of these 256 sites, 210 were among the 242 detected prior to adjusting for neuron proportion thus suggesting that cell composition does not account for the majority of observed differences in DNAm.

Genomic regions in which DNAm at multiple contiguous sites differs between SZ and NPC subjects, or DMRs, may be more biologically meaningful or have different functional consequences than those of a single DMS. There were 44 genomic regions in which DNAm at two or more contiguous, measured sites differed between subjects with SZ and NPC subjects (Table 3).

**Table 3:** Differentially methylated regions in SZ.

Chromosome	Start	End	Length in bp	Number of significant probes	Mean *β* value coefficient	Overlapping promoters	Overlapping genes
6	28828946	28829503	557	15	0.012562318	LINC01623, RPL13P	LINC01623, RPL13P
1	16062361	16063471	1110	11	−0.010632242	SLC25A34, RP11-288I21.1	SLC25A34, RP11-288I21.1
1	3191219	3192542	1323	10	−0.021685336		PRDM16
10	6263235	6264776	1541	9	−0.016688209	PFKFB3	PFKFB3
19	48958216	48959043	827	9	−0.014250403	KCNJ14	GRWD1
15	93617080	93617833	753	9	−0.016227133	RGMA	RGMA
1	2999586	3001128	1542	8	−0.020050418		PRDM16
4	1800154	1801294	1140	8	−0.012881307	FGFR3	FGFR3
3	187453721	187454786	1065	8	−0.015068722	BCL6	BCL6
3	13082751	13083684	933	7	−0.018337881		IQSEC1
1	153600597	153600972	375	7	−0.016748303	S100A1, S100A13	S100A1, S100A13
3	12949457	12950513	1056	6	−0.015753296		IQSEC1
4	1803298	1805090	1792	6	−0.015286045	FGFR3	FGFR3
8	141588118	141588943	825	6	−0.017229636		AGO2
2	8711042	8711256	214	6	−0.018511245		LINC01814
4	1807819	1808646	827	6	−0.01256232	FGFR3	FGFR3
4	866299	866811	512	6	−0.017785773	GAK	GAK
14	21491808	21492316	508	6	−0.010876489	NDRG2	NDRG2
10	99329961	99330447	486	5	−0.014591035	ANKRD2	UBTD1
2	241535685	241536284	599	5	−0.021733676	CAPN10	CAPN10
1	3154081	3154700	619	5	−0.023621906	PRDM16	PRDM16
5	1033518	1034076	558	4	−0.023946075	NKD2	NKD2
19	45411802	45412647	845	4	−0.023104719		APOE
7	2262244	2262479	235	4	−0.020981488	MAD1L1	MAD1L1
6	5087031	5087749	718	4	−0.021469791	PPP1R3G	PPP1R3G, LYRM4
9	138966848	138967347	499	4	−0.021449114		NACC2
10	7212815	7213304	489	4	−0.021980366		SFMBT2
12	117157450	117158154	704	4	−0.015498341	C12orf49	C12orf49
10	3162054	3162191	137	4	−0.017789142		PFKP
13	79170146	79170430	284	4	0.014049989		OBI1-AS1
9	96199463	96199581	118	3	−0.02791665	RP11-165J3.6-001	
17	55703709	55704098	389	3	−0.029515629		MSI2
5	167858238	167858326	88	3	−0.017722017	WWC1-008, WWC1-007	WWC1
22	47016623	47017522	899	3	−0.020100319	GRAMD4	GRAMD4
17	79686849	79687075	226	3	−0.026164519		SLC25A10
1	223945127	223945297	170	3	−0.022033434	CAPN2	CAPN2
1	3128606	3129115	509	3	−0.024216925		PRDM16
15	93611859	93611950	91	2	−0.024325293		RGMA
7	4829256	4829350	94	2	−0.028189732	AP5Z1, MIR4656	AP5Z1
15	70364327	70364359	32	2	−0.020032632		TLE3
15	65594642	65594648	6	2	−0.024798094	PARP16	RP11-349G13.2
14	65689711	65689836	125	2	−0.021050816	LINC02324	
17	77954963	77955167	204	2	−0.0173864		TBC1D16
3	58558333	58558343	10	2	−0.018019101	RP11-475O23.2	FAM107A

The 44 genomic regions in which DNAm differed at two or more contiguous, measured sites between SZ and NPC subjects are listed.

*DNAm* DNA methylation, *DMR* differentially methylated region, *SZ* schizophrenia, *NPC* non-psychiatric comparison, *bp* base pairs.

Notably, three DMSs and one DMR were identified within the mitotic arrest deficient 1-like 1 (*MAD1L1*) gene. *MAD1L1* is one of 130 genes thought likely to explain the association between SNPs at 270 GWAS loci and SZ [3]. The *MAD1L1*-associated differential methylation we identified was located in exon 6, and the methylation levels were lower in SZ subjects relative to NPC subjects.

### SZ-associated differential DNA methylation at some individual sites was specific to neurons or glia

**Fig. 2:**
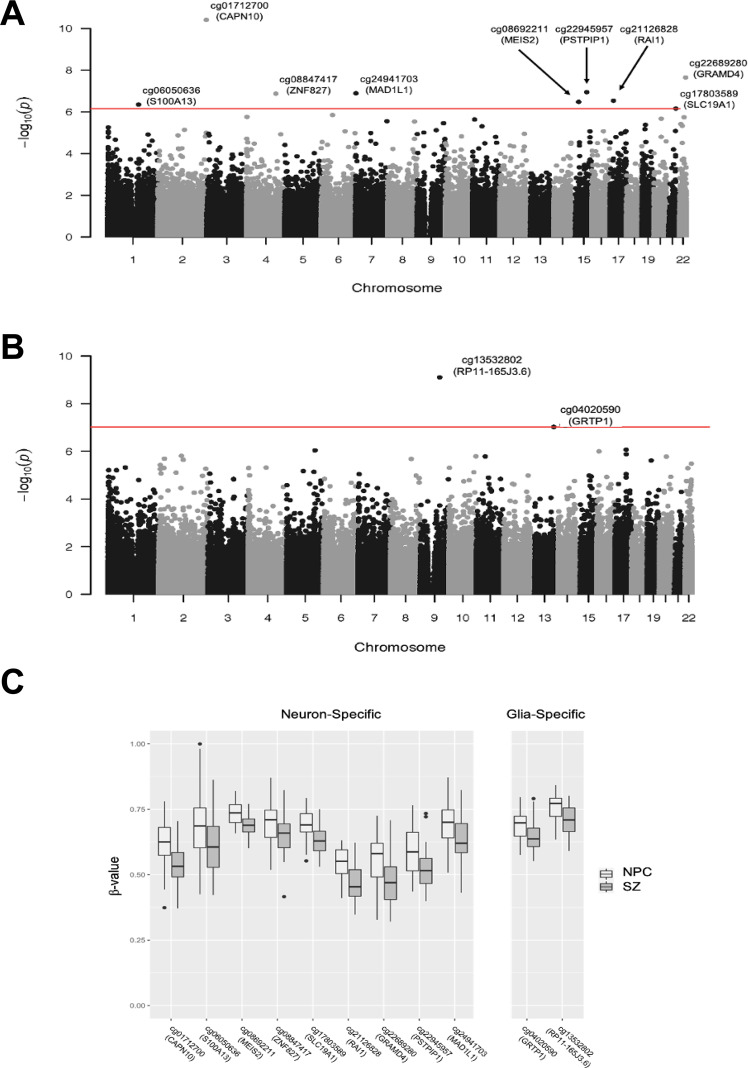
Neuron- or glia-specific differential methylation in SZ. **A** Manhattan plot showing neuron-specific DNAm differences between SZ and NPC subjects at nine sites. **B** Manhattan plot showing glia-specific DNAm differences between SZ and NPC subjects at two sites. **C** Box plots of DNAm (*β* value) at sites of cell-type-specific differences in DNAm between SZ and NPC subjects. DNAm DNA methylation, SZ schizophrenia, NPC non-psychiatric comparison.

Cell-type deconvolution identified nine DMSs in neurons (Fig. 2A, C) and two DMSs in glia (Fig. 2B, C) with an FDR cutoff of *q* = 0.1. One of the sites of SZ-associated differential methylation identified within *MAD1L1* in bulk tissue analysis was determined to be due to neuron-specific DNAm alterations by cell-type deconvolution. (Fig. 2B, C). All 11 sites for which DNAm differed between SZ and NPC subjects in a cell-type-specific manner were also identified as being differentially methylated in the bulk tissue analysis (Table 2).

### Brain methylation and transcript quantitative trait loci for *MAD1L1* and schizophrenia GWAS signals are colocalized at the *MAD1L1*-containing locus

**Fig. 3:**
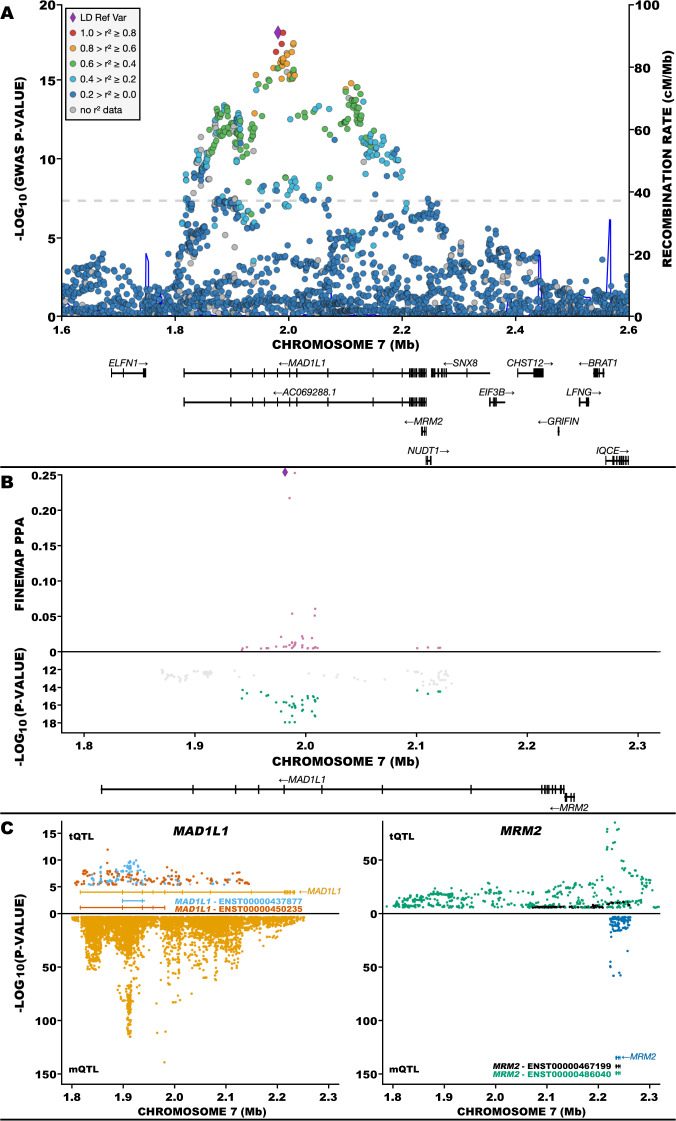
Fine mapping and colocalization analysis at *MAD1L1*-containing locus. **A** Negative log (base 10) *p* values for association at chromosome 7p22.3 from a genome-wide association study (GWAS) of schizophrenia [3]. The purple diamond represents SNP rs12668848 (*p* = 1.110 × 10^−18^). Not shown: insertion/deletion variants. **B** Mirror plot of fine-mapping posterior probability (PPA; upper plot) and SZ associations (from **A**) at chromosome 7p22.3 (lower plot). In the upper plot, the largest PPA was 0.254 (purple diamond). The remaining points are PPA computed on SZ-GWAS SNPs with association *p* < 5 × 10^−15^. The lower plot shows SNPs with SZ association *p* <5 × 10^−12^. The green points represent SNPs used for fine mapping because they have SZ association *p* < 5 × 10^−15^; SNPs not shown have negligible PPA. **C** Mirror plot of transcript quantitative trait loci (tQTL; upper plots) and methylation quantitative trait loci (mQTL; lower plots) for *MAD1L1* (left) and *MRM2* (right). The tQTLs and mQTLs were obtained from PsychENCODE [76] and Jaffe et al. [7], respectively. For *MAD1L1* (upper left), 95 and 165, out of a total of 262 tQTL SNPs mapped to transcripts ENST00000437877 (teal points) and ENST00000450235 (red points), respectively. For *MRM2* (upper right), 154 and 545, out of a total of 699 tQTL SNPs mapped to transcripts ENST00000467199 (black points) and ENST00000480040 (green points), respectively. In the lower half of each plot, mQTLs are depicted that show 11,368 mQTL SNPs mapping to 280 CpG sites associated with *MAD1L1* (yellow points; lower left) and 129 mQTL SNPs mapping to 4 CpG sites associated with *MRM2* (blue points; lower right).

Based on the fine-mapping of the GWAS signal at the *MAD1L1*-containing locus (Fig. 3A), no single SNP stood out as the causal variant (all had PPA <0.5); moreover, there was no support for more than one causal variant at this locus either (Fig. 3B and Supplementary Table 3A). Many mQTLs and tQTLs fell within this locus (Fig. 3C), with the tQTLs affecting the expression of three transcripts mapping onto two genes (Fig. 3C).

Joint analysis of GWAS and tQTL data for three transcripts yielded PPA = 0.95, 0.74, and 0.29 for ENST00000437877 (*MAD1L1*), ENST00000450235 (*MAD1L1*), and ENST00000486040 (*MRM2*), respectively (Supplementary Table 3B). By the convention that PPA >0.8 is sufficient evidence for colocalization, ENST00000437877 (*MAD1L1*) was colocalized at rs58120505. Joint analysis of GWAS, tQTL, and mQTL data also provided compelling evidence of colocalization for two *MAD1L1* transcripts (Supplementary Table 3C); for ENST00000437877, again the greatest evidence accrued at rs58120505. Diagnostics for these colocalizations were imperfect (Supplementary Fig. 4), however; this might be explained by an imperfect match between the etiological effect of the causal genetic variant and the tissue used to produce the tQTL and mQTL resources.

Our results comported with a recent study by Perzel Mandell et al. [36], who used whole-genome bisulfite sequencing to assess DNAm in 183 subjects (344 samples) of human postmortem brain tissue, as well as characterize the genome-wide genetic variation of all subjects. In the Perzel Mandell study, two brain regions were characterized, the hippocampus and the dorsolateral prefrontal cotex. Using the signal from a GWAS study of SZ [35], they selected an index SNP to represent the GWAS signal in each locus (usually the SNP with the smallest *p* value). They found that these index SNPs were highly likely to be mQTLs. Their index SNP for the GWAS signal around *MAD1L1*, rs10650434, was no exception; it was a significant mQTL, associating with almost 2,000 CpG sites in the locus regardless of a brain region, although the strongest mQTL signals, as judged by *p* value, were for CpG sites within a few kilobases (kb) of the SNP itself. Notably, the index SNP, rs10650434, lies within 5 kb of the SNP we colocalized, rs58120505, and alleles of the two SNPs are in almost perfect linkage disequilibrium (*r*^2^ = 0.992), according to genotypes from 498 samples of European ancestry reported in the 1000 Genomes Project [37].

## Discussion

In the STG of SZ subjects, we identified differences in DNAm levels relative to NPC subjects at 242 individual sites and 44 genomic regions with multiple sites. Notably, we identified SZ-associated differential methylation in *MAD1L1*, a gene contained within one of the 270 SZ risk loci identified in the largest GWAS study to date and one of 130 genes thought highly likely to explain the association between GWAS loci and SZ [3]. The *MAD1L1*- associated differential methylation we identified was characterized by lower DNAm in SZ subjects relative to NPC subjects, a difference we determined to be driven by neuron-specific alterations in DNAm. This finding is consistent with studies in the prefrontal cortex that also identified genome-wide significant DMRs in *MAD1L1* [7]. Using publicly available data, we identified brain mQTLs and tQTLs for *MAD1L1* and found evidence for colocalization with the GWAS signal at the *MAD1L1*-containing locus.

Our findings, and those of Perzel Mandell et al. [36], implicate *MAD1L1* methylation in SZ etiology and/or pathophysiology, and suggest alterations in *MAD1L1* methylation and transcription may mediate SZ risk at the *MAD1L1*-containing locus. Despite pointing to a potential molecular mechanism by which SZ risk SNPs at the *MAD1L1*-containing locus confer risk, the biological mechanisms affected by *MAD1L1*-associated SZ risk variants and differential methylation that might be relevant to conferring SZ risk remain unclear. *MAD1L1* is expressed in many human tissues [38, 39] and is known to have a role in regulating the spindle assembly checkpoint during mitosis [40]. Genetic mutations that disrupt *MAD1L1* expression are associated with aneuploidy and multiple cancers [38, 39]. During development, *MAD1L1* is most strongly expressed in differentiating cells and is critical for the transition from proliferation to terminal differentiation in a broad range of cell types [41, 42]. Given *MAD1L1* is expressed in both neurons and glia of most brain regions [43–45], the differentiation of neurons and glia may be disrupted if *MAD1L1* expression is affected by SZ-associated differential methylation during neurodevelopment. Such a disruption would be predicted to alter the delicate balance of the various neuronal and glia subtypes and thus brain circuitry, perhaps giving rise to the dysfunctional brain circuits that are associated with the clinical features of SZ [46].

Alternatively, *MAD1L1* may act post-neurodevelopment as its expression in terminally differentiated cells, including post-mitotic neurons and glia, suggests a function in addition to those related to development. Studies have found that its expression in terminally differentiated cells may be necessary for maintaining the differentiated state [47–49]. Indeed, even modest decreases in *MAD1L1* expression lead to dedifferentiation in some cell types [48]. Some evidence points to a role for dedifferentiation of post-mitotic neurons in the cognitive decline and behavioral changes associated with normal brain aging in humans [50–52], and a similar mechanism could conceivably contribute to SZ etiology and/or pathophysiology. That said, these proposed mechanisms are conjecture and critical next steps should focus on understanding *MAD1L1* in the brain, generally, and translating *MAD1L1*-associated SNPs and differential methylation into molecular mechanisms for SZ, specifically.

This study is the first to identify SZ-associated differential methylation in the STG. Others have previously reported DNAm differences between subjects with SZ and NPC subjects in the prefrontal cortex [7, 53–56], striatum [55], hippocampus [55, 57], and cerebellum [55] thus suggesting that altered DNAm in multiple brain regions contributes to SZ neurobiology. Our findings add to the growing body of literature that implicates altered epigenetic pathways, including DNAm as well as histone modifications [58–60], in SZ neurobiology. Though most often studied separately, there is extensive crosstalk between DNAm and histone modification pathways [61–63]. This crosstalk drives the establishment of composite epigenetic signatures that depend on epigenetic regulatory enzymes (e.g., DNA methyltransferases, histone methyltransferases, etc.) with protein domains that specifically recognize methylated DNA and/or modified histones and thus allow for linking of DNAm and histone modifications at appropriate sites in the genome. SETD1A, a gene in which loss-of-function mutations confer a large increase in risk for SZ [64], is an example of an enzyme linking DNAm and histone modification. SETD1A methylates histones after it is localized to unmethylated DNA via an interaction with CXXC-finger protein-1 [65]. This body of literature suggests that a more complete understanding of how these epigenetic pathways and their interactions are altered in SZ is likely to be fruitful in identifying molecular mechanisms contributing to SZ. Epigenetic editing technologies that use highly specific DNA-targeting tools (e.g., CRISPR) to methylate DNA or modify histones in a locus-specific manner will be valuable in dissecting these molecular mechanisms in cell culture and animal models [66, 67].

Though our findings for MAD1L1 strongly implicate genetic variation as a causal mechanism for its SZ-associated differential methylation, our findings of other DMSs in this study, like those of all postmortem brain studies, are only correlative and cannot establish causal relationships. The SZ-associated differences in DNAm that we identified in the STG are likely a combination of genetic and environmental factors [68–70].

Though differential methylation may be associated with SZ risk factors, it may be the result of exposure to antipsychotics or other confounds. Studies of peripheral tissues indicate that antipsychotics do alter DNAm [71]. However, DNAm alterations are already present in subjects with only brief (<16 weeks) antipsychotic treatment [72], thus suggesting that much SZ-associated differential methylation is intrinsic to the illness. Some studies have even found that SZ-associated DNAm alterations in peripheral tissues are normalized by treatment with antipsychotics [73], raising the possibility that the therapeutic effects of antipsychotics are mediated, in part, by DNAm changes. Such findings also make it likely that antipsychotics mask some SZ-associated differential methylation from being detected in studies.

An additional potential confound particularly relevant in studies of DNAm in subjects with SZ is cigarette smoking. Cigarette smoking is much more common among individuals with SZ than the general population and is known to induce robust DNAm changes in peripheral tissues [74]. Cigarette smoking does affect DNAm in the brain, however, none of the DMSs or regions identified in this study have been found to be among the sites most strongly affected by cigarette smoking [75].

This study lays the groundwork for more detailed investigations of SZ-associated differential methylation in the STG. Future studies should focus on identifying the biological mechanisms by which altered DNAm, especially within *MAD1L1*, contributes to SZ etiology and pathophysiology. To this end, studies that use epigenetic editing technology to recapitulate SZ-associated differential methylation in cell cultures and animal models will be useful.

## Supplementary information

Supplementary Methods

Supplementary Figure 1

Supplementary Figure 2

Supplementary Figure 3

Supplementary Figure 4

Supplementary Table 1

Supplementary Table 2A

Supplementary Table 2B

Supplementary Table 3
