# Supplementary material for: Schizophrenia-associated differential DNA methylation in brain is distributed across the genome and annotated to MAD1L1, a locus at which DNA methylation and transcription phenotypes share genetic variation with schizophrenia risk
Source: Transl Psychiatry. 2022 Aug 20;12:340. doi: 10.1038/s41398-022-02071-0 (PMC9392724; doi:10.1038/s41398-022-02071-0)
Supplement: Supplementary file 1 — Supplementary Methods [file 41398_2022_2071_MOESM1_ESM.docx]

## SUPPLEMENTAL METHODS:

*RELATING DNA METHYLATION AND GENE TRANSCRIPTION TO GWAS SIGNAL AT THE MAD1L1-CONTAINING SZ RISK LOCUS*

Fine Mapping and Colocalization Analyses. To understand the likely etiological cause of the SZ association signals mapping to 7p22.3, we performed fine mapping and colocalization analyses. We started by fine mapping the SZ GWAS signal obtained by Pardiñas and colleagues ^1^. These results were visualized using Locuszoom (Figure 3; GRCh38/hg38 used for all analyses); however, insertion/deletion variants were excluded from this plot – but not other analyses – because the index variant was an indel and the Locuszoom program had no linkage disequilibrium (LD) information about this SNP ^2^.

Fine Mapping**.** To localize the SZ association signal in 7p22.3 to specific SNPs, we used the Bayesian fine-mapping algorithm FINEMAP ^3^. Flanking SNP genotypes that were not available were determined using LD patterns (n=496 European founders reference panel from the 1000 Genomes and r > 0.81; ^4^). After determining these genotypes, there were 43 SNPs used for fine mapping that had a minimum SZ GWAS p < 5×10^-15^. The FINEMAP algorithm then evaluated numerous possible causal variant configurations, producing posterior probabilities of association (PPA) that a given DNA variant was the causal variant. Notably, while no variant emerged as the clear causal variant from this analysis, because all PPA<0.50 (Figure 3B; Supplemental Table 3A), results from FINEMAP clearly favor only one causal SNP at this locus (data not shown).

Colocalization Analyses. Next, we probed this region for joint colocalization of the SZ GWAS and quantitative trait loci (QTL) specific to gene transcripts (tQTL; PsychENCODE; ^5^). Using the PsychENCODE tQTL data, we identified 262 and 699 tQTLs for *MAD1L1* and *MRM2*, respectively. Of these, 95 and 165 tQTLs, out of a total of 262 tQTLs, mapped to *MAD1L1* transcripts ENST00000437877 and ENST00000450235, respectively. These tQTL SNPs were then analyzed for colocalization of GWAS and transcript expression signal using MOLOC ^6^. In the *MAD1L1* locus, we detected colocalization with transcript ENST00000437877 (PPA=0.95; Figure 3C; Supplemental Figure 4A). Another MAD1L1 transcript (ENST00000450235) approached the customary threshold (0.80) for declaring colocalization (PPA=0.74; Figure 3C; Supplemental Figure 4B; Supplemental Table 3B).

For the *MRM2* gene, we also observed highly significant tQTLs (Figure 3C; Supplemental Figure 4C). Notably, however, the peak tQTL signal, whose -log_10_ p-value > 10^-80^, was approximately 200 kilobases (kb) away from the peak SZ GWAS signal (Figure 3A). Given the massive signal for this index SNP and the LD patterns in the GWAS locus, we wondered if the tQTL signal observed for all other SNPs in this locus could be explained solely by the signal at the index SNP. To evaluate this possibility, we used the method detailed in DIST^7^ to impute the expected z-score for all other regional SNPs based on the z-score at the index SNP and on the LD patterns from 1000G founder subjects of European ancestry. The fit between the imputed and observed z-scores was almost perfect (Supplemental Figure 4D; R^2^ = 0.955), showing that the tQTL signal at all other SNPs arises from the index SNP. This was important because the index SNP was not included in the set of fine-mapped SNPs; moreover, for any SNP near the GWAS index SNP and showing *MRM2* tQTL signal, its signal was merely a reflection of the *MRM2* index SNP, which is far away from GWAS signal. For these reasons, any SNP that showed some evidence for colocalization of the GWAS signal and signal for *MRM2*, and there are some, were not true colocalizations. Such SNPs were ignored by our analysis. Indeed, when we evaluated diagnostics for colocalization, *MRM2* showed extremely poor fit (Supplemental Figure 4C); the fits for *MAD1L1* transcripts were better, but their fits also were not ideal (Supplemental Figures 4A and 4B).

We next mined the mQTL data by Jaffe and colleagues^8^ to identify overlapping mQTLs in this region. There was a total of 11,368 mQTLs that mapped to 280 CpG sites associated with *MAD1L1* and 129 mQTLs that mapped to four CpG sites associated with *MRM2*. The mapping of CpG sites to genes follows resource http://zwdzwd.github.io/InfiniumAnnotation#download ^9^, after lift over to hg38. These data allowed us to colocalize, using MOLOC, the GWAS signal using two other types of information, tQTL and mQTL. We limited this analysis to the two aforementioned *MAD1L1* transcripts. Because a predefined region was a requirement for the MOLOC analysis, we required the following conditions for colocalization analysis: each CpG site was assessed plus an additional flanking 20 kb (40 kb in total), combinations of tQTLs and mQTLs that were associated with *MAD1L1*, and at least two SNPs were within each 40 kb region for which we had the full information on SZ GWAS, tQTL, and mQTL statistics. (MOLOC requires at least two SNPs for colocalization.) Notice that this colocalization analysis was CpG-centric and, because we utilized the Jaffe data, we used their definition for cis SNPs (+/- 20 kb). Given these conditions, 197 CpG sites met these criteria (Supplemental Table 3C). Within these CpG sites, there were 66 unique SNPs that had a PPA>0.80. Furthermore, there were 44 CpG sites that contained ten or more SNPs and had at least one SNP with PPA>0.80. These results, mapping onto 19 unique SNPs, were displayed in Figure 3D: 29 CpG sites for which SNPs mapped to *MAD1L1* transcript ENST00000467877; and 15 CpG sites for which SNPs mapped to *MAD1L1* transcript ENST00000450235.

## SUPPLEMENTAL METHODS REFERENCES:

1 Pardiñas, A. F., Holmans, P., Pocklington, A. J., Escott-Price, V., Ripke, S., Carrera, N. *et al.* Common schizophrenia alleles are enriched in mutation-intolerant genes and in regions under strong background selection. *Nature Genetics*, doi:10.1038/s41588-018-0059-2 (2018).

2 Boughton, A. P., Welch, R. P., Flickinger, M., VandeHaar, P., Taliun, D., Abecasis, G. R. *et al.* LocusZoom.js: Interactive and embeddable visualization of genetic association study results. *Bioinformatics*, doi:10.1093/bioinformatics/btab186 (2021).

3 Benner, C., Spencer, C. C. A., Havulinna, A. S., Salomaa, V., Ripatti, S. & Pirinen, M. FINEMAP: Efficient variable selection using summary data from genome-wide association studies. *Bioinformatics* **32**, doi:10.1093/bioinformatics/btw018 (2016).

4 Genomes Project, C., Auton, A., Brooks, L. D., Durbin, R. M., Garrison, E. P., Kang, H. M. *et al.* A global reference for human genetic variation. *Nature* **526**, 68-74, doi:10.1038/nature15393 (2015).

5 Psych, E. C., Akbarian, S., Liu, C., Knowles, J. A., Vaccarino, F. M., Farnham, P. J. *et al.* The PsychENCODE project. *Nat Neurosci* **18**, 1707-1712, doi:10.1038/nn.4156 (2015).

6 Giambartolomei, C., Liu, J. Z., Zhang, W., Hauberg, M., Shi, H., Boocock, J. *et al.* A Bayesian framework for multiple trait colocalization from summary association statistics. *Bioinformatics*, doi:10.1093/bioinformatics/bty147 (2018).

7 Lee, D., Bigdeli, T. B., Riley, B. P., Fanous, A. H. & Bacanu, S. A. DIST: direct imputation of summary statistics for unmeasured SNPs. *Bioinformatics* **29**, 2925-2927, doi:10.1093/bioinformatics/btt500 (2013).

8 Jaffe, A. E., Gao, Y., Deep-Soboslay, A., Tao, R., Hyde, T. M., Weinberger, D. R. *et al.* Mapping DNA methylation across development, genotype and schizophrenia in the human frontal cortex. *Nat Neurosci* **19**, 40-47, doi:10.1038/nn.4181 (2016).

9 Zhou, W., Laird, P. W. & Shen, H. Comprehensive characterization, annotation and innovative use of Infinium DNA methylation BeadChip probes. *Nucleic Acids Res* **45**, e22, doi:10.1093/nar/gkw967 (2017).
