# Supplementary material for: Schizophrenia-associated differential DNA methylation in brain is distributed across the genome and annotated to MAD1L1, a locus at which DNA methylation and transcription phenotypes share genetic variation with schizophrenia risk
Source: Transl Psychiatry. 2022 Aug 20;12:340. doi: 10.1038/s41398-022-02071-0 (PMC9392724; doi:10.1038/s41398-022-02071-0)
Supplement: Supplementary file 2 — Supplementary Figure 1 [file 41398_2022_2071_MOESM2_ESM.pdf]

Supplemental Figure 1

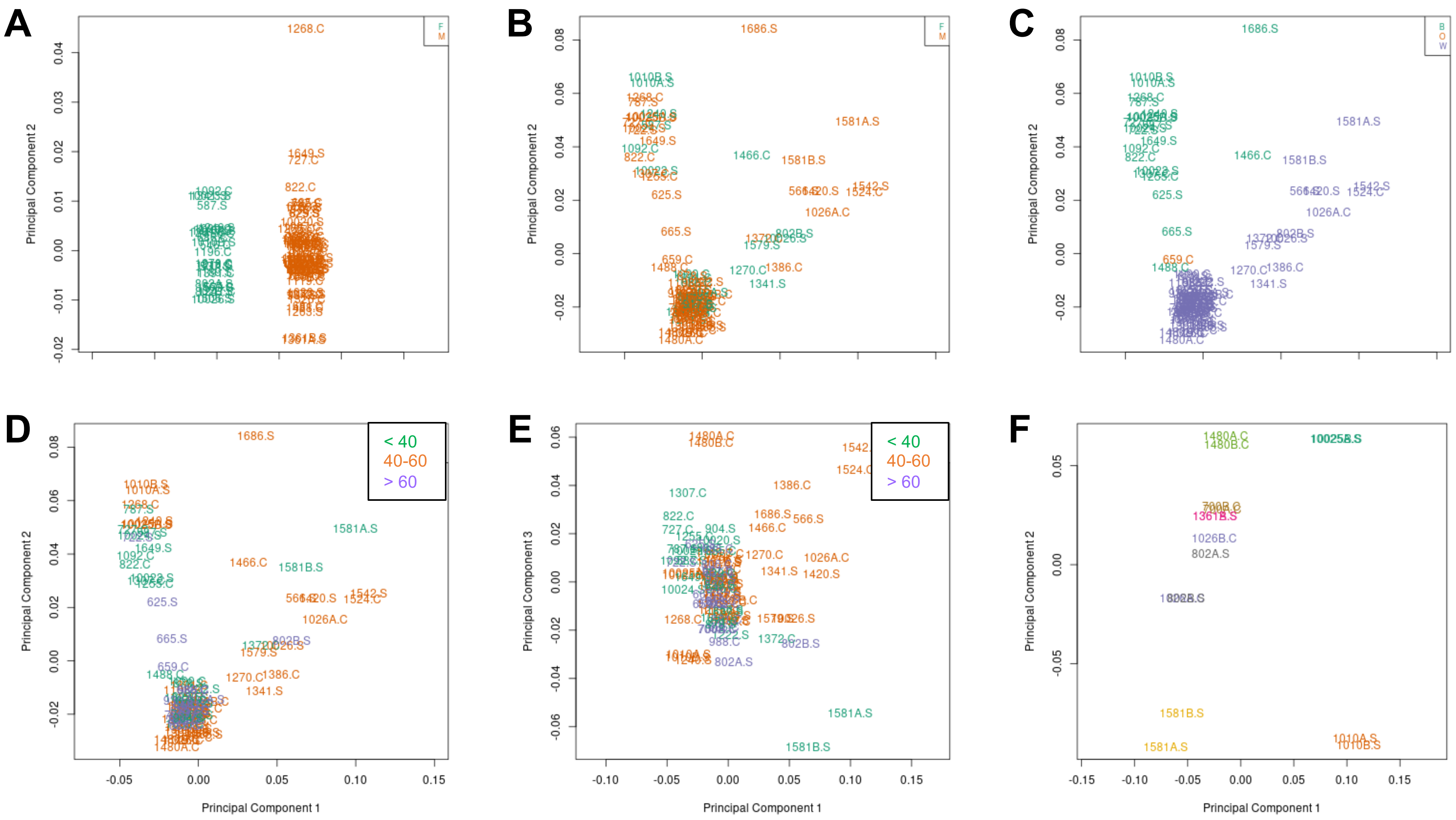

Supplemental Figure 1. Mutidimensional scaling (MDS) visualization of similarity among subjects based on EPIC array data. Prior to data filtering, subjects were distinctly segregated by sex **(A)**. After data filtering, MDS using data from the 3000 most variable sites was performed and samples no longer segregated by sex **(B)**, but segregation by race **(C)** and age **(D, E)** became evident. The replicate sample pairs from each of the eight subjects from which replicate samples were collected and assayed co-segregated in MDS space **(F)**. S, schizophrenia; C, non-psychiatric comparison; M, male; F, female; B, black; O, other (Asian Indian); W, white.
