# Supplementary figures and images for: Schizophrenia-associated differential DNA methylation in brain is distributed across the genome and annotated to MAD1L1, a locus at which DNA methylation and transcription phenotypes share genetic variation with schizophrenia risk
Source: Transl Psychiatry. 2022 Aug 20;12:340. doi: 10.1038/s41398-022-02071-0 (PMC9392724; doi:10.1038/s41398-022-02071-0)

## Supplemental Figure 2

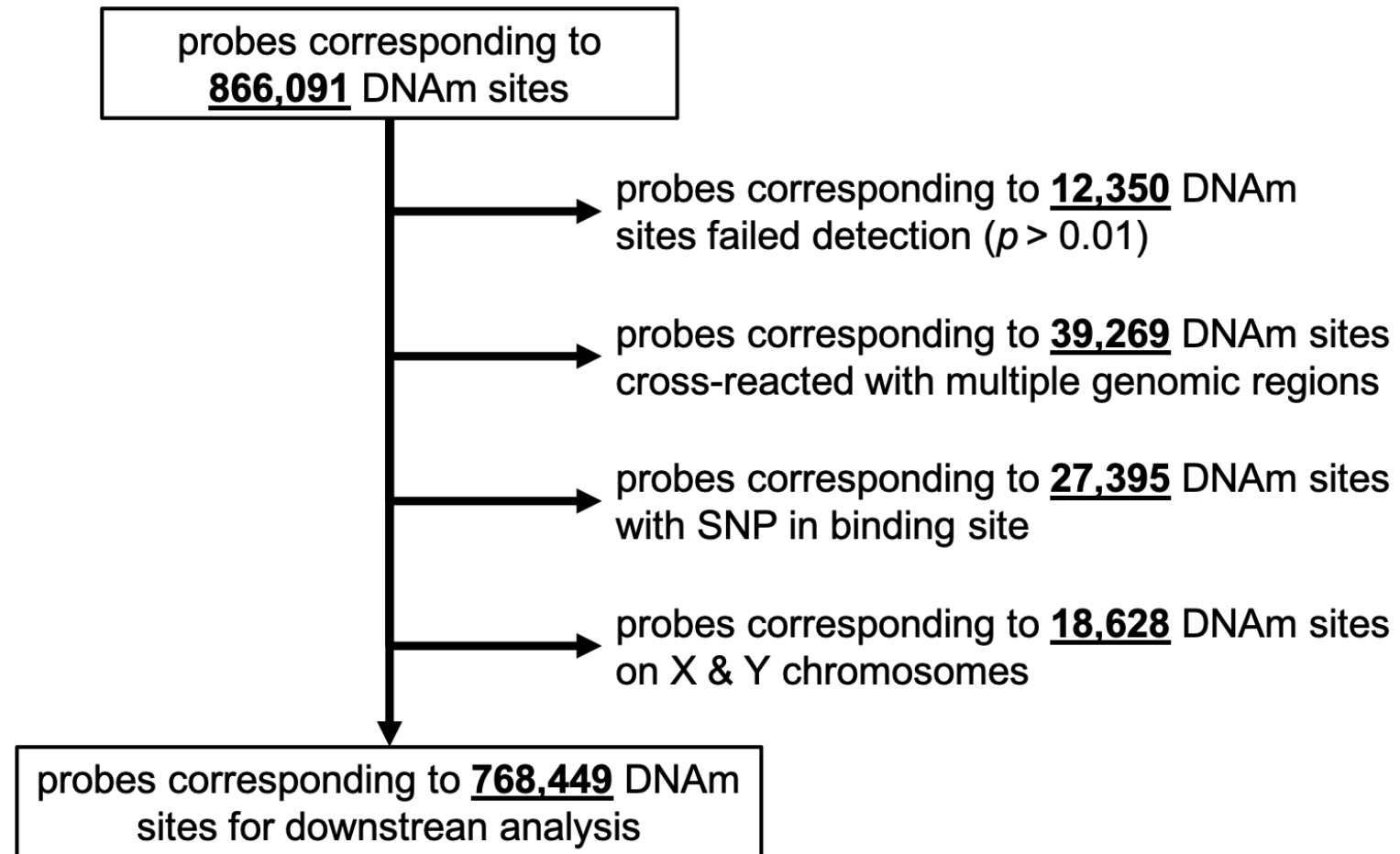

Supplemental Figure 2. Schematic representation of probe filtering.

Supplement: Supplementary file 3 — Supplementary Figure 2 [file 41398_2022_2071_MOESM3_ESM.pdf]
