# Supplementary material for: Schizophrenia-associated differential DNA methylation in brain is distributed across the genome and annotated to MAD1L1, a locus at which DNA methylation and transcription phenotypes share genetic variation with schizophrenia risk
Source: Transl Psychiatry. 2022 Aug 20;12:340. doi: 10.1038/s41398-022-02071-0 (PMC9392724; doi:10.1038/s41398-022-02071-0)
Supplement: Supplementary file 4 — Supplementary Figure 3 [file 41398_2022_2071_MOESM4_ESM.pdf]

Supplemental Figure 3

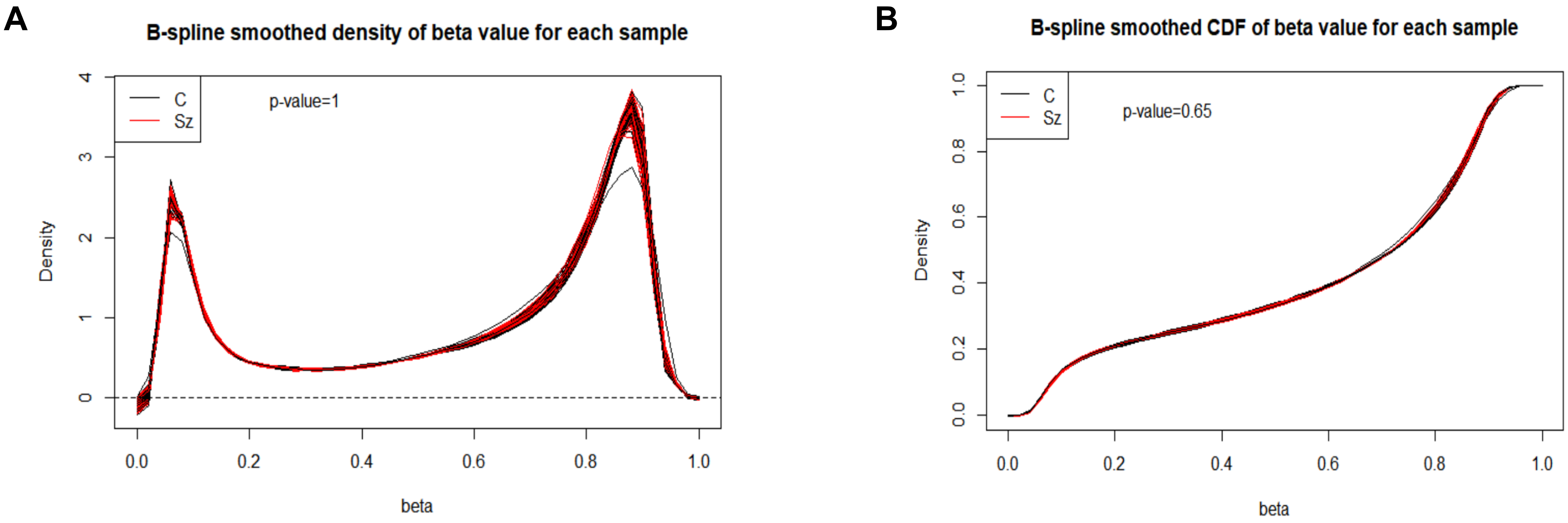

Supplemental Figure 3. Global DNA methylation in schizophrenia and non-psychiatric comparison subjects. Global DNA methylation does not differ between schizophrenia and non-psychiatric comparison subjects as assessed by comparison of B-spline smoothed **(A)** density distributions (SKAT-based test, $p>0.99$) and **(B)** cumulative density functions (CDF). (SKAT-based test, $p=0.65$). Sz, schizophrenia; C, non-psychiatric comparison.
