# Supplementary material for: Schizophrenia-associated differential DNA methylation in brain is distributed across the genome and annotated to MAD1L1, a locus at which DNA methylation and transcription phenotypes share genetic variation with schizophrenia risk
Source: Transl Psychiatry. 2022 Aug 20;12:340. doi: 10.1038/s41398-022-02071-0 (PMC9392724; doi:10.1038/s41398-022-02071-0)
Supplement: Supplementary file 5 — Supplementary Figure 4 [file 41398_2022_2071_MOESM5_ESM.pdf]

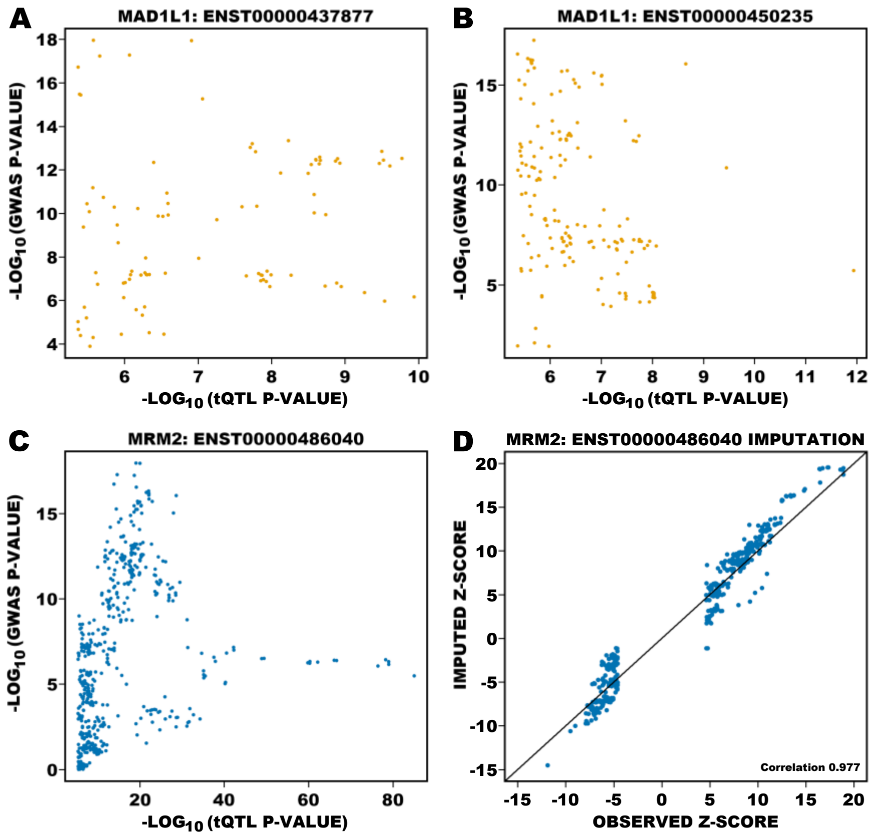

Supplemental Figure 4. Colocalization Diagnostic Plots. (**A**, **B**) Genome wide association study (GWAS) of schizophrenia (3) sites were plotted against transcript quantitative trait loci (tQTL; PsychEncode; 73) sites for two *MAD1L1* transcripts (yellow) and (**C**) one *MRM2* transcript (blue). (**D**) Z-Score imputation for *MRM2* transcript.
