# Supplementary material for: Schizophrenia-associated differential DNA methylation in brain is distributed across the genome and annotated to MAD1L1, a locus at which DNA methylation and transcription phenotypes share genetic variation with schizophrenia risk
Source: Transl Psychiatry. 2022 Aug 20;12:340. doi: 10.1038/s41398-022-02071-0 (PMC9392724; doi:10.1038/s41398-022-02071-0)
Supplement: Supplementary file 6 — Supplementary Table 1 [file 41398_2022_2071_MOESM6_ESM.pdf]

Supplemental Table 1. Characteristics of human subjects. Abbreviations: W, white; B, black; O, other (Asian Inidan); M, male; F, female; NPC, non-psychiatric comparison; SZ, schizophrenia; PMI, postmortem interval; ASCVD, athrosclerotic cardiovascular disease; CDO, combined drug overdose; GI, gastrointestinal; MVP, mitral valve prolapse; PE, pulmonary embolism**.**

Brain Bank ID	Age	Race	Sex	Diagnostic Group	Cause of Death	Manner of Death	PMI (hours)
1326	58	W	M	NPC	ASCVD	Natural	16.4
1247	58	W	F	NPC	ASCVD	Natural	22.7
1086	51	W	M	NPC	ASCVD	Natural	24.2
1201	52	W	F	NPC	ASCVD	Natural	16.4
1255	37	B	M	NPC	PE	Natural	22.0
1480	62	W	M	NPC	ASCVD	Natural	20.1
1119	57	W	M	NPC	ASCVD	Natural	20.2
1317	56	W	M	NPC	ASCVD	Natural	22.9
1307	32	B	M	NPC	ASCVD	Natural	4.8
1067	49	W	M	NPC	ASCVD	Natural	6.0
1196	36	W	F	NPC	Asphyxiation	Accidental	14.5
1099	24	W	F	NPC	Cardiomyopathy	Natural	9.1
806	57	W	M	NPC	PE	Natural	24.0
739	40	W	M	NPC	ASCVD	Natural	15.8
822	28	B	M	NPC	ASCVD	Natural	25.3
727	19	B	M	NPC	Trauma	Accidental	7.0
659	46	O	M	NPC	Peritonitis	Accidental	22.2
852	54	W	M	NPC	Cardiac Tamponade	Natural	8.0
685	56	W	M	NPC	Hypoplastic coronary artery	Natural	14.5
686	52	W	F	NPC	ASCVD	Natural	22.6
1092	40	B	F	NPC	MVP	Natural	16.6
1488	39	B	M	NPC	PE	Natural	21.5
1047	43	W	M	NPC	ASCVD	Natural	13.8
700	42	W	M	NPC	ASCVD	Natural	26.1
567	46	W	F	NPC	MVP	Natural	15.0
871	28	W	M	NPC	Trauma	Accidental	16.5
988	82	W	M	NPC	Trauma	Accidental	22.5
1284	55	W	M	NPC	ASCVD	Natural	6.4
1555	17	W	M	NPC	Trauma	Accidental	15.1
1268	49	B	M	NPC	ASCVD	Natural	19.9
1466	64	B	F	NPC	Trauma	Accidental	20.0
1386	46	W	M	NPC	ASCVD	Natural	21.2
1026	59	W	M	NPC	ASCVD	Natural	19.8

1524	66	W	M	NPC	Small intestinal infarction	Natural	9.4
1270	73	W	F	NPC	Trauma	Accidental	19.7
1372	37	W	M	NPC	Asphyxiation	Accidental	20.5
1374	43	W	M	NPC	ASCVD	Natural	21.7
1159	51	W	M	NPC	ASCVD	Natural	16.7
857	48	W	M	NPC	ASCVD	Natural	16.6
1391	51	W	F	NPC	ASCVD	Natural	7.8
818	67	W	F	NPC	Anaphylactic reaction	Accidental	24.0
630	65	W	M	NPC	ASCVD	Natural	21.2
546	37	W	F	NPC	ASCVD	Natural	23.5
681	51	W	M	NPC	Hypertrophic cardiomyopathy	Natural	11.6
1453	62	W	M	SZ	Trama	Accidental	11.1
1240	50	B	F	SZ	ASCVD	Natural	22.9
10025	52	B	M	SZ	ASCVD	Natural	27.1
1256	34	W	M	SZ	Hanging	Suicide	27.4
1189	47	W	F	SZ	CDO	Suicide	14.4
10020	38	W	M	SZ	Salicylate overdose	Suicide	28.8
1263	62	W	M	SZ	Asphyxiation	Accidental	22.7
625	49	B	M	SZ	ASCVD	Natural	23.5
1173	62	W	M	SZ	ASCVD	Natural	22.9
1361	63	W	M	SZ	Cardiomyopathy	Natural	23.2
10024	37	B	M	SZ	ASCVD	Natural	6.0
1296	48	W	M	SZ	Pneumonia	Natural	7.8
1211	41	W	F	SZ	Sudden unexpected death	Natural	20.1
10023	25	B	F	SZ	Drowning	Suicide	20.1
665	59	B	M	SZ	Intestinal hemorrhage	Natural	28.1
1088	49	W	M	SZ	CDO	Accidental	21.5
787	27	B	M	SZ	Gunshot	Suicide	19.2
829	25	W	M	SZ	Salicylate overdose	Suicide	5.0
930	47	W	M	SZ	ASCVD	Natural	15.3
722	45	B	M	SZ	Upper GI bleed	Natural	9.1
1105	53	W	M	SZ	ASCVD	Natural	7.9
802	63	W	F	SZ	Right ventricular dysplasia	Natural	29.0
1010	44	B	F	SZ	Sudden unexpected death	Natural	18.7
1222	32	W	M	SZ	CDO	Suicide	30.8

933	44	W	M	SZ	Myocarditis	Natural	8.3
917	71	W	F	SZ	ASCVD	Natural	23.8
537	37	W	F	SZ	Hanging	Suicide	14.5
878	33	W	M	SZ	Myocardial fibrosis	Natural	10.8
621	83	W	M	SZ	Asphyxiation	Accidental	16.0
1188	58	W	M	SZ	ASCVD	Natural	7.7
1649	17	B	M	SZ	Hanging	Suicide	21.4
1230	50	W	M	SZ	Doxepin overdose	Suicide	16.9
1341	44	W	F	SZ	Trauma	Accidental	24.5
1420	47	W	M	SZ	Jumping	Suicide	23.4
1542	65	W	M	SZ	CDO	Undetermined	17.4
566	63	W	M	SZ	ASCVD	Natural	18.3
587	38	B	F	SZ	Myocardial hypertrophy	Natural	17.8
1579	69	W	F	SZ	ASCVD	Natural	16.1
1581	32	W	M	SZ	ASCVD	Natural	18.4
10026	46	W	F	SZ	Thermal injuries	Suicide	23.8
1686	56	B	M	SZ	ASCVD	Natural	14.1
904	33	W	M	SZ	Bronchopneumonia	Natural	28.0
1455	42	W	M	SZ	Peritonitis	Natural	8.2
1506	47	W	F	SZ	CDO	Accidental	14.1
