# Supplementary material for: Schizophrenia-associated differential DNA methylation in brain is distributed across the genome and annotated to MAD1L1, a locus at which DNA methylation and transcription phenotypes share genetic variation with schizophrenia risk
Source: Transl Psychiatry. 2022 Aug 20;12:340. doi: 10.1038/s41398-022-02071-0 (PMC9392724; doi:10.1038/s41398-022-02071-0)
Supplement: Supplementary file 7 — Supplementary Table 2A [file 41398_2022_2071_MOESM7_ESM.pdf]

Supplemental Table 2A. Neuronal proportion in SZ and NPC subjects. Neuron proportion did not differ between STG samples from SZ and NPC subjects (two-sample t-test; p=0.50). Abbreviations: NPC, non-psychiatric comparison; STG, superior temporal gyrus; SZ, schizophrenia;

**Neuron Proportion**	**SZ**	**NPC**
Median (minimum-maximum)	0.45 (0.38-0.61)	0.46 (0.37-0.59)
Median (standard deviation)	0.46 (0.04)	0.46 (0.05)
