# Supplementary material for: Schizophrenia-associated differential DNA methylation in brain is distributed across the genome and annotated to MAD1L1, a locus at which DNA methylation and transcription phenotypes share genetic variation with schizophrenia risk
Source: Transl Psychiatry. 2022 Aug 20;12:340. doi: 10.1038/s41398-022-02071-0 (PMC9392724; doi:10.1038/s41398-022-02071-0)
Supplement: Supplementary file 8 — Supplementary Table 2B [file 41398_2022_2071_MOESM8_ESM.pdf]

Supplemental Table 2B. Differentially methylated sites in SZ after adjusting for neuronal proportion. The 256 sites at which DNAm differs between SZ and NPC subjects at the FDR cutoff of q=0.1 (adjusted for age, race, PMI, and neuronal proportion) are listed in the table. Abbreviations: DNAm, DNA methylation; FDR, false discovery rate; NPC, non-psychiatric comparison; PMI, postmortem interval; SZ, schizophrenia

Site	DNAm Difference (SZ-NPC; β-values)	q-value	Gene
cg01712700	-0.032	8.39E-05	CAPN10
cg13532802	-0.041	9.36E-05	
cg02113493	-0.022	0.005	
cg04020590	-0.033	0.007	GRTP1
cg25079492	-0.028	0.011	CLEC16A
cg05621596	-0.018	0.012	GRAMD4
cg08847417	-0.023	0.012	ZNF827
cg23453794	-0.033	0.012	MERTK
cg08692211	-0.021	0.018	MEIS2
cg23379913	-0.026	0.018	AKAP1
cg07748741	-0.012	0.019	UBTD1
cg24941703	-0.027	0.019	MAD1L1
cg26981306	-0.027	0.019	KIAA0892
cg04011474	-0.028	0.020	
cg22945957	-0.027	0.020	PSTPIP1
cg22519912	-0.027	0.026	PSD2
cg01606571	-0.026	0.026	HADHA
cg02478836	-0.021	0.026	TBC1D22A
cg06050636	-0.034	0.026	S100A13
cg13913915	-0.031	0.026	MSI2
cg18735473	-0.020	0.026	BRUNOL4
cg21620968	-0.029	0.026	COPS7B
cg22348992	-0.016	0.026	CHRNA4
cg22689280	-0.028	0.026	
cg02289653	-0.025	0.027	EPB41L1
cg10863857	-0.024	0.027	CELF2
cg12349571	-0.017	0.027	TLE3
cg13295422	-0.030	0.027	CELF2; CELF2-AS2
cg14346040	-0.018	0.027	NR1D1
cg19261949	-0.023	0.027	ZMAT5
cg21265339	-0.022	0.027	
cg24158028	-0.027	0.027	STK32C
cg18282392	-0.019	0.029	GALNT7
cg25459000	-0.017	0.031	SYNGR1
cg19418922	-0.026	0.031	EXT2
cg03449456	-0.020	0.031	PRDM16
cg02722031	-0.027	0.032	HERC3

cg08425757	-0.013	0.032	TRAPPC9
cg12937501	-0.025	0.033	
cg14608424	-0.025	0.033	ABR
cg17601209	-0.031	0.033	PRDM16
cg00387200	-0.027	0.035	
cg09200437	-0.018	0.035	
cg10699522	-0.028	0.035	DST; LOC101930010
cg15044372	-0.023	0.035	
cg23200394	-0.033	0.035	GLI2
cg24920126	-0.022	0.035	PPP1R3G
cg12446793	-0.028	0.036	
cg24883899	-0.018	0.036	APC2
cg07348768	-0.014	0.036	PRDM16
cg16901627	-0.019	0.036	COPE
cg25894668	-0.021	0.040	SLC3A2
cg13523224	-0.031	0.043	CFAP99
cg20402747	-0.019	0.044	TBC1D16
cg24512544	-0.020	0.044	EIF2C2
cg25211200	-0.025	0.044	MRVI1
cg21408848	-0.026	0.046	IQSEC1
cg24318537	-0.023	0.046	UNC119B
cg06022867	-0.043	0.047	
cg12449974	-0.027	0.047	CORO2B
cg01203812	-0.026	0.048	PRDM16
cg12713481	-0.027	0.048	
cg12590902	-0.022	0.048	ERI3
cg18329758	-0.027	0.048	WWC1
cg21946195	-0.033	0.050	ATOH8
cg00772497	-0.027	0.050	GSTCD
cg13689085	-0.026	0.050	TCF7
cg00159552	-0.018	0.051	TBC1D22A
cg00162902	-0.019	0.051	FAM184A
cg00686823	-0.024	0.051	TPRA1
cg01123449	-0.026	0.051	HHIPL1
cg01287037	-0.027	0.051	
cg01433955	-0.027	0.051	
cg02347483	-0.021	0.051	CCDC101
cg03595140	-0.027	0.051	FNBP1
cg03932760	-0.022	0.051	ARRB1; MIR326
cg04500745	-0.017	0.051	MAPK8IP3
cg04633409	0.019	0.051	TWF1
cg05068943	-0.019	0.051	GOT1
cg05501958	-0.011	0.051	APOE
cg06317803	0.018	0.051	
cg06847567	0.020	0.051	

cg07380086	-0.025	0.051	CHN1
cg07597386	-0.007	0.051	PRDM16
cg07605200	-0.026	0.051	
cg08196145	-0.018	0.051	
cg08209664	-0.020	0.051	ST3GAL1
cg08256119	-0.025	0.051	MSI2
cg08419879	-0.017	0.051	PLEKHG1
cg09815962	-0.026	0.051	EIF2C2
cg09925572	-0.025	0.051	TFCP2
cg11197533	-0.026	0.051	IFT122
cg12312611	0.027	0.051	ARRDC5
cg13136596	-0.034	0.051	MSI2
cg14020176	-0.020	0.051	SLC9A3R1
cg14366878	0.025	0.051	SLC1A1
cg14372037	-0.029	0.051	SORCS2
cg14517390	-0.019	0.051	ACSBG1
cg15165927	-0.023	0.051	NKD2
cg15728120	-0.017	0.051	CENPT
cg15748271	-0.020	0.051	TRIM8
cg15845746	-0.012	0.051	TMEM177
cg16266918	-0.024	0.051	PDXK
cg16433632	-0.013	0.051	RAMP1
cg17134838	-0.022	0.051	
cg17803589	-0.023	0.051	SLC19A1
cg19788036	-0.025	0.051	
cg21785920	-0.038	0.051	LBP
cg22548266	-0.027	0.051	SPOCK2
cg22649529	-0.033	0.051	TECR
cg23634532	-0.022	0.051	OGDH
cg24338094	-0.022	0.051	PLXNA1
cg25298833	-0.027	0.051	RGMA
cg25601830	-0.022	0.051	AKR7A2
cg26201596	0.026	0.051	
cg26520908	-0.028	0.051	PRDM16
cg26580673	-0.020	0.051	
cg26632239	-0.014	0.051	CTDP1
cg27151770	-0.021	0.051	ZNF423
cg27214458	-0.013	0.051	MRGPRF; MRGPRF-AS1
cg21705669	-0.017	0.052	BCL6
cg08213909	-0.026	0.054	MCC
cg12128274	0.030	0.054	CNOT4
cg16023894	-0.029	0.054	EPHB2
cg16622899	0.020	0.054	MAFK
cg26409376	-0.032	0.054	
cg07987705	-0.022	0.054	RGMA

cg18783374	-0.022	0.054	MSI2
cg16011164	-0.027	0.056	MIR4656; AP5Z1
cg17529670	-0.022	0.056	BCR
cg14297573	-0.032	0.058	PFKP
cg09255521	-0.034	0.058	
cg01952185	0.019	0.059	
cg04677723	-0.017	0.062	MOGS
cg04618897	-0.028	0.066	KIAA0415
cg07303829	-0.020	0.066	SAPS2
cg11548242	-0.018	0.066	
cg11569621	-0.019	0.066	
cg13821176	-0.029	0.066	TRIB1
cg19736604	-0.023	0.066	TNXB
cg22014398	-0.019	0.066	MGAT4B
cg24484600	-0.021	0.066	GDPD5
cg09788030	-0.024	0.067	
cg02752163	0.045	0.068	
cg03629926	-0.019	0.068	ANGPTL4
cg03649589	-0.018	0.068	CSGALNACT1
cg07463740	-0.025	0.068	
cg16187038	-0.023	0.068	RCOR1
cg21126828	-0.026	0.068	RAI1
cg25580656	-0.024	0.068	ZFYVE21
cg23879743	-0.018	0.068	
cg11301187	-0.025	0.068	KIAA0195
cg05321174	-0.014	0.068	PTK2B
cg26301507	-0.018	0.068	SLC25A20
cg13259703	-0.022	0.070	
cg20108328	-0.023	0.070	C21orf70
cg19859445	-0.022	0.070	
cg04792024	-0.023	0.070	TMEM120A
cg07504768	-0.021	0.070	MTSS1L
cg23564627	-0.022	0.070	PEMT
cg24379495	-0.021	0.073	SLC1A2
cg12985235	-0.010	0.073	MPND
cg14335434	-0.032	0.073	PRKCH
cg05071292	-0.013	0.074	LOC728613
cg05927518	-0.021	0.074	TACC1
cg15343406	-0.037	0.074	
cg17196564	-0.020	0.074	
cg19177744	-0.023	0.074	
cg21036560	-0.024	0.075	PGBD5
cg03628962	-0.021	0.075	RGMA
cg03957687	-0.023	0.075	CENPT
cg04594439	-0.025	0.075	PASK

cg07018888	0.017	0.075	FOXN1
cg09214323	-0.023	0.075	RNU6-2; KIF1B
cg13175786	-0.021	0.075	PRDM16
cg17282060	-0.012	0.075	ARHGAP22
cg17736422	-0.033	0.075	PRDM16
cg24986651	-0.024	0.075	LPIN1
cg26000554	-0.023	0.075	MOSC2
cg26913155	-0.026	0.075	PRDM16
cg15365305	-0.021	0.076	SMARCA2
cg26122413	-0.019	0.076	INF2
cg05141465	-0.026	0.078	CHST10
cg10225499	-0.031	0.079	EZR
cg17320669	-0.026	0.080	CAPN2
cg19839415	-0.022	0.080	IFFO2
cg03950655	-0.020	0.084	ROR1
cg22029806	-0.018	0.084	CD47
cg00634968	0.022	0.085	PKHD1
cg01053681	-0.026	0.085	ZMIZ1
cg01118541	0.019	0.085	
cg03747028	0.011	0.085	TAF12
cg04964562	-0.016	0.085	PLCG1
cg05526364	-0.026	0.085	IFT122
cg05878289	-0.023	0.085	SORCS2
cg06520014	-0.016	0.085	
cg07077978	-0.027	0.085	
cg15412087	-0.023	0.085	OAF
cg16229161	-0.019	0.085	TNK2
cg25119743	-0.024	0.085	CUGBP2
cg00415704	-0.025	0.086	RGMA
cg00726470	-0.016	0.086	
cg01800445	-0.035	0.086	PRDM16
cg12863924	-0.026	0.086	
cg13153666	-0.022	0.086	
cg18069081	-0.024	0.086	GPR39
cg03272941	-0.019	0.086	RHOJ
cg17823326	-0.016	0.086	NUBPL
cg24186251	-0.023	0.086	SH3RF3
cg26915329	0.023	0.086	
cg02986801	-0.019	0.086	ST3GAL1
cg26051775	-0.019	0.087	CAPN2
cg25307778	-0.021	0.088	ERI1
cg00813147	0.019	0.088	AMOTL2
cg15763706	-0.026	0.089	SRGAP3
cg08589214	-0.023	0.089	CAPN10
cg12441066	-0.025	0.089	MSI2

cg14597213	-0.021	0.089	AHCYL1
cg20988960	-0.030	0.089	PRDM16
cg25619978	0.016	0.089	TRPC7
cg19055270	0.059	0.089	
cg02133510	-0.022	0.091	TNXB
cg05026881	0.011	0.091	TPSG1
cg05112420	-0.023	0.091	BCR
cg05747038	-0.019	0.091	GLIS3
cg07714715	-0.026	0.091	DENND1A
cg13691436	-0.023	0.091	FRMD4A
cg14229175	-0.038	0.091	IMPA2
cg22177068	-0.028	0.091	ATP13A4-AS1; ATP13A4
cg22738000	-0.014	0.091	RASSF4
cg25456772	0.016	0.091	RAB3IP
cg13302567	-0.025	0.093	MAD1L1
cg04844692	-0.020	0.093	C12orf49
cg25122824	-0.017	0.093	MAD1L1
cg26489368	-0.022	0.093	NKD2
cg07580832	-0.021	0.094	MSI2
cg08067895	-0.023	0.094	CDX1
cg09185640	-0.018	0.094	CORO2B
cg09509365	-0.020	0.094	PRDM16
cg09761288	-0.023	0.094	
cg09792192	-0.017	0.094	AHCYL2
cg13547132	-0.019	0.094	
cg16326073	-0.019	0.094	PRR5
cg07410783	-0.024	0.096	CLEC16A
cg14583999	-0.030	0.096	TMEM111
cg17876641	-0.025	0.096	KIF21B
cg00305491	-0.026	0.096	WWC1
cg05005073	-0.034	0.097	
cg05318384	0.021	0.098	
cg08441850	0.030	0.098	IKZF3
cg05912181	-0.018	0.098	LOC100506497
cg13904892	-0.018	0.098	C15orf62; DNAJC17
cg01419991	-0.022	0.098	TRIB1
cg05808227	-0.023	0.099	
cg02743070	-0.018	0.099	ZMIZ1
cg24699097	-0.016	0.100	RAB11FIP4
